# Exceptional visuospatial imagery in schizophrenia; implications for madness and creativity

**DOI:** 10.3389/fnhum.2013.00756

**Published:** 2013-11-11

**Authors:** Taylor L. Benson, Sohee Park

**Affiliations:** Clinical Neuroscience Laboratory, Department of Psychology, Vanderbilt UniversityNashville, TN, USA

**Keywords:** schizophrenia, creativity, mental imagery, parietal lobe, working memory

## Abstract

Biographical and historical accounts suggest a link between scientific creativity and schizophrenia. Longitudinal studies of gifted children indicate that visuospatial imagery plays a pivotal role in exceptional achievements in science and mathematics. We asked whether visuospatial imagery is enhanced in individuals with schizophrenia (SZ). We compared SZ and matched healthy controls (HC) on five visuospatial tasks tapping parietal and frontoparietal functions. Two aspects of visuospatial transformation, spatial location and mental imagery manipulation were examined with Paper Folding Test (PFT) and jigsaw puzzle task (JPT), respectively. Visuospatial intelligence was assessed with Ravens Progressive Matrices, which is associated with frontoparietal network activity. Hemispatial inattention implicating parietal function was assessed with line bisection (LB) task. Mediated by prefrontal cortex, spatial delayed response task (DRT) was used to index working memory maintenance, which was impaired in SZ compared to HC. In contrast, SZ showed intact visuospatial intelligence and transformation of location. Further, SZ performed significantly better than HC on JPT indicating enhanced mental imagery manipulation. Spatial working memory (SWM) maintenance and mental imagery manipulation were strongly associated in HC but dissociated in SZ. Thus, we observed enhanced mental imagery manipulation in SZ but the dissociation of mental imagery from working memory suggests a disrupted frontoparietal network. Finally, while HC showed the expected leftward pseudoneglect, SZ showed increased rightward LB bias implicating left hemispatial inattention and impaired right parietal control of spatial attention. The current results chart a unique profile of impaired, spared and enhanced parietal-mediated visuospatial functions implicating parietal abnormalities as a biobehavioral marker for SZ. We discuss these results in relation to creative cognition.

## Introduction

“Everything you can imagine is real.”—Pablo Picasso

Two large-scale, longitudinal studies of gifted children in the past half-century have sought to answer the question of what lies at the core of exceptional abilities. Project TALENT (http://www.projecttalent.org/) began in 1960 with a total sample size of over 440,000 adolescents who were given tests designed to assess a wide range of cognitive abilities including mathematical, verbal, and spatial (see Wai et al., 2009). Similarly, the Study of Mathematically Precocious Youth (SMPY; Lubinski and Benbow, 2006) began in early 1970s and has been tracking mathematically talented children (top 0.5%) in several waves of cohorts (Wai et al., 2010). One striking finding that emerged from these studies is the prominent role of visuospatial ability (rather than mathematical ability) in predicting subsequent achievements and eminence in science, technology, engineering and mathematics (Wai et al., 2010; Kell et al., 2013). The spatial abilities tested in these studies involved mental imagery such as visualization of two and three-dimensional objects and manipulation of mental representations (e.g., mental rotation).

Mental images are the building blocks of human consciousness that comprise one's internal representation of the world. Historical and biographical accounts have often suggested the pivotal role of mental imagery in exceptional creativity. For instance, Albert Einstein rarely thought in words, preferring instead to rely on images (Wertheimer, 1945; Shepard and Cooper, 1982; Miller, 1989, 1992, 2000).

“Words or language … do not seem to play any role in my mechanism of thought … my elements of thought are … images” (Albert Einstein)

Einstein's reliance on imagery is believed to have played a major role in his thought processes and discoveries. Indeed, Einstein's special theory of relativity had its roots in a thought experiment that he had been visualizing since his adolescence. As a boy, he wanted to know what a beam of light would look like if he could ride alongside it. So he imagined himself traveling at the speed of light with a mirror held in front of him. In this imaginary scenario, he could never see his reflection in the mirror since the light and the mirror were traveling at the same velocity and the mirror was always held a little ahead of the beam. Such use of mental images while working on new theories mark Einstein's thinking style.

Much has been speculated about the nature and source of Einstein's genius. Initial analyses of his brain structures had not yielded anything remarkable but the recent discovery of lost slides of Einstein's brain and subsequent re-analyses of cortical region by Falk et al. (2013) provide a fascinating glimpse into the possible neural basis of his extraordinary mental imagery. Generation, inspection, and manipulation of visuospatial mental images involve parietal and frontal cortical networks (Kosslyn et al., 2001; Slotnick et al., 2012). Compared with 85 other brains, three features of Einstein's cortical structures stood out as extraordinary. Einstein had a very unusual and intricate pattern of convolution in the prefrontal cortex bilaterally. Prefrontal cortex is involved in working memory, planning, meta-cognition, counterfactual thinking and simulation, allowing us to control and manipulate mental representations to guide behavior (see Fuster, 2008). Mental representations or imagery is an essential part of simulation and it enables us to use internal representations to play out what could happen in hypothetical situations. Thus, mental imagery offers an opportunity to visualize the many potential consequences of a decision, operation, or action.

A second unique feature of Einstein's brain was the asymmetry and the size of his parietal cortex, which plays a central role in visuospatial functions, especially mental imagery, mathematical ability and multisensory processing. Einstein's right superior parietal lobule (SPL) was strikingly different from his left SPL. The third unusual aspect of his brain involves the sensory motor cortices. An area of the motor cortex that processes information from the face, tongue, and larynx was expanded into a large rectangular patch that apparently has not been observed in other brains (see Falk et al., 2013, Figure 10). Falk and colleagues quote Einstein as describing his thoughts as an association of images and “feelings,” and that these thoughts were also “muscular” in addition to visual. Is it possible that the peculiarities of his brain allowed him to see, feel, touch, and move in his thoughts? While these structural anomalies cannot explain Einstein's genius, it nevertheless begs the question of the role of visuospatial mental imagery in extraordinary talent and giftedness.

An important footnote to the case of Einstein is his genetic liability for schizophrenia as a first-degree relative. Genetic or psychometric high-risk for schizophrenia has been linked to elevated creativity in epidemiological (Kyaga et al., 2011), behavioral (Nelson and Rawlings, 2010) and neuroimaging studies (Folley and Park, 2005). Einstein's youngest child, Eduard, was diagnosed with schizophrenia at the age of twenty while studying medicine and subsequently spent his life in and out of the University Psychiatric Clinic in Zürich (Burghölzli), sometimes in the care of Professor Manfred Bleuler. While Einstein did not suffer from any psychotic illnesses, his risk for schizophrenia would have been elevated 10-fold as a first-degree relative of the proband (Cardno and Gottesman, 2000), and more importantly, functional neuroimaging data indicate that the patients and their first-degree relatives share very similar patterns of functional connectivity (Whitfield-Gabrieli et al., 2009).

These autobiographical anecdotes are confirmed by research (Forisha, 1978; Roskos-Ewoldson et al., 1993; LeBoutillier and Marks, 2003). In adults, high correlations have been found between the use and control of mental images and divergent thinking (Durndell and Wetherick, 1976; Forisha, 1981), ideational fluency, and learning associated name pairs (Hargreaves and Bolton, 1972). Khatena (1973, 1978, 1983) found that originality in thinking was associated with the tendency to process complex mental images. Finally, the vividness of mental images is related to creative thinking skills (Forisha, 1981; Campos and Gonzalez, 1995). In theory, one might think that the vividness of the mental image and the ability to transform mental images is related to creativity because they both rely on the manipulation and flexible combination of figural representations, but the relationship is unclear.

A creative idea must be both novel and appropriate (Sternberg and O'Hara, 1999). The eminent ideas put forth by Einstein certainly fit this profile. The ability to mentally visualize and manipulate the elements of a scenario may be essential to creative pursuits (Arp, 2011). Recent research identifies possible neural correlates of visuospatial creativity that might suggest an association between enhanced creativity and mental imagery in creative individuals such as Einstein (Aziz-Zadeh et al., 2013). The DLPFC was activated bilaterally in a visuospatial creativity task, showing an increase in left DLPFC activation when compared to a control mental imagery task. Motor areas including the SMA, inferior frontal gyrus and premotor cortex were also preferentially activated in the left hemisphere. The mental imagery task recruited the right inferior parietal lobule known to be involved in mental rotation, while the visuospatial creativity task showed a significant increase in the left inferior parietal lobule activation suggesting increased bilateral activation in creative cognition (Aziz-Zadeh et al., 2013). The brain regions active during visuospatial creativity are analogous to the structures that were increased in density and convolution in Einstein's brain. It is also interesting to note that these regions share many overlapping similarities with the mirror neuron network (Rizzolatti and Craighero, 2004). Additionally, Einstein's left inferior parietal lobule and right SPL were unusually large and may have been responsible in part for his increased creative and spatial abilities, respectively.

In this paper, we examine the link between mental imagery (MI), creativity and psychopathology to move beyond these fascinating anecdotal case studies and show why we need neuroscience to understand the origins of human creativity. Previous research has emphasized the relationship between mental imagery and at least two fundamental features of schizophrenia (SZ), a severely debilitating psychotic disorder characterized by widespread cognitive impairments that negatively impact functional outcome (Green et al., 2000). Specifically, mental imagery is closely linked with hallucinations, a core diagnostic symptom of schizophrenia (APA, 2000), and working memory, an endophenotype candidate for the disorder (Glahn et al., 2003).

Working memory is an active, limited-capacity, short-term memory system, which temporarily maintains information, and provides an interface between perception, long-term memory and action (Baddeley, 1992). Temporary maintenance of information is supported by a supervisory attentional control system called the “central executive” and modality-specific buffers (phonological loop, visuospatial sketchpad) that feed into a multimodal “episodic storage buffer” with a capacity limit of about four chunks. Deployment of attentional resources, selection of control processes/strategies and coordination of information flow from the sub-systems are mediated by the central executive. Thus, the top–down cognitive control by the central executive and bottom-up perceptual input interact to keep the mental representation alive in working memory. It is important to note that working memory consists of multiple neurocognitive components and processes, of which some may be intact while others are impaired in schizophrenia. There are multiple types of working memory, including verbal working memory, visual working memory, and spatial working memory (SWM). Verbal working memory allows us to manipulate verbal (auditory) information, while visual working memory enables individuals to manipulate visual information (Luck and Vogel, 1997). SWM is a subdivision of visual working memory that specifically deals with spatial information about the visual input.

Patients with schizophrenia consistently demonstrate impaired performance on behavioral tasks requiring the utilization of SWM ability (Park and Holzman, 1992; see Lee and Park, 2005 for a meta-analysis). Previous neuroimaging studies have provided evidence that impaired SWM performance is associated with abnormal cortical activation along frontoparietal pathways (Barch and Csernansky, 2007; Lee et al., 2008). Importantly, this impairment in SWM ability is present in SZ patients regardless of clinical state (Park et al., 1999), in addition to individuals at high-risk for schizophrenia, including first-degree relatives (e.g., Park et al., 1995; Glahn et al., 2003), and can be detected in the schizophrenia-spectrum across different sensory modalities (Lee and Park, 2005).

In contrast to the cognitive deficits observed in the schizophrenia spectrum, recent research indicates that SZ patients show spared abilities in certain cognitive domains, and sometimes even enhanced performance when compared to healthy controls (HC). Understanding these rare enhancements in schizophrenia is important since they may lead to a more complete understanding of the complex cognitive profile of patients with schizophrenia, rather than just describing a generalized deficit in cognitive performance. Such enhancements include self-reported vividness of mental imagery (e.g., Sack et al., 2005), mental rotation (Thakkar and Park, 2012), and the generation, inspection and manipulation of mental images (Matthews et al., 2013). Thus, it is interesting to note that almost all of the few reported enhancements found in SZ patients involve mental imagery.

Mental images are similar to actual percepts in terms of their functions in interference tasks, image scanning times, and brain activation patterns (Kosslyn, 1980). Crucially, mental images are intentionally generated and manipulated in the absence of external stimuli (Kosslyn, 1980). Indeed, the intention, agency, and control of these internal representations distinguish mental imagery from the similar process of hallucination (Bentall, 1990). In other words, while mental images and hallucinations are both similar in experience to actual percepts, the main difference between the two is that mental imagery may be intentionally produced and controlled by the individual, whereas hallucinations are not under one's control or conscious awareness. Several researchers have investigated the connection between vividness of mental imagery and hallucinations (e.g., Mintz and Alpert, 1972; Silbersweig and Stern, 1998; Bocker et al., 2000; Aleman et al., 2003; Oertel et al., 2009), but the evidence for this hypothesis (i.e., that increased vividness of mental imagery is linked to hallucinatory experiences) has been mixed in the literature and is thus far inconclusive. In fact, enhanced vividness of mental imagery in schizophrenia has been hypothesized as a trait marker for schizophrenia (e.g., Sack et al., 2005; Oertel et al., 2009), after obtaining results indicating that enhanced mental imagery appears to be ubiquitous among patients with schizophrenia (i.e., independent of clinical symptoms, including hallucinations).

Since both mental imagery and working memory have been hypothesized to be markers for schizophrenia (i.e., enhanced mental imagery as a trait marker and impaired working memory as an endophenotype candidate), it is interesting that these two cognitive processes show opposite patterns of results (i.e., enhanced mental imagery and impaired working memory). The puzzle becomes even more complex in light of the realization that mental imagery and working memory are both cognitive processes that rely on the maintenance and manipulation of internal representations (Kosslyn, 1980). In healthy participants, SWM and mental imagery are highly correlated (Baddeley and Andrade, 2000), as they are able to successfully utilize their visuospatial sketchpad to facilitate their memory (Baddeley, 1992). However, individual differences appear to modulate the relationship between imagery and working memory (Gur and Hilgard, 1975; Constantinidis and Wang, 2004; Keogh and Pearson, 2011). Thus, it is noteworthy that SZ patients are impaired in working memory despite their superior mental imagery performance when working memory is usually supported by imagery in healthy people.

Despite this intriguing dissociation, however, only a handful of studies have empirically investigated the dissociation between mental imagery and working memory in SZ patients. The few that have attempted such a feat in the past have mostly focused on parsing out maintenance vs. manipulation components of the SWM deficit. Thakkar and Park (2012) investigated active manipulation of MI using two mental rotation tasks and compared performance on these tasks with that on a passive SWM task in patients with SZ. They found that while SWM maintenance was impaired in patients with SZ, the same individuals showed superior performance on a mental rotation task compared with matched HC. The authors concluded that SZ patients exhibit impaired passive maintenance of internal representations but show evidence of intact ability to manipulate mental imagery (Thakkar and Park, 2012).

Similarly, Matthews et al. (2013) recently found mental imagery manipulation enhancement in SZ patients compared to HC using an imagery generation and inspection task (see Zarrinpar et al., 2006). Yet in a subsequent experiment testing both maintenance and manipulation, these researchers found that the additional maintenance component in the new task caused the imagery manipulation enhancement displayed by the SZ patients in the previous study to disappear. To be clear, by passive maintenance it is meant that individuals must simply maintain information over a delay period. As such, the authors concluded that SZ patients can show intact or even enhanced performance on tasks requiring the use of manipulation of mental imagery, but as soon as working memory maintenance component is added to the task (and subsequent cognitive load of the patient), this MI manipulation enhancement is masked in SZ patients (Matthews et al., 2013).

One potential benefit that might result from enhanced mental imagery includes increased creative achievement, which has been found to be linked with the schizophrenia-spectrum (Abraham et al., 2005; Folley and Park, 2005; Abraham et al., 2007), particularly in SZ patients' first-degree relatives (e.g., Karlsson, 1970, 1984), and has been found to be related to enhanced mental imagery in HC (LeBoutillier and Marks, 2003). Ever since Huxley et al. (1964) presented the idea of a “schizophrenia paradox” in an effort to explain the stable prevalence rate of schizophrenia in the population despite reduced fecundity (Larson and Nyman, 1973) and increased mortality (Brown, 1997), there have been many attempts to explain a compensatory advantage for the genes associated with schizophrenia (Hasenfus and Magaro, 1976; Brüne, 2004; Pearlson and Folley, 2008).

One possibility is the presence of pockets of enhanced creativity in probands of the disorder (O'Reilly et al., 2001; Nettle and Clegg, 2006). The connection between creativity and psychosis has been speculated for centuries and reinforced by the romantic idea that many well known creative individuals may have been afflicted with psychological illness (Post, 1994). While most of the early studies documenting this link are based superficially on biographical data (Andreasen and Powers, 1975; Jamison, 1993; Post, 1994; Weisberg, 1994), recent research regarding enhanced creativity in SZ has utilized empirical, experimental paradigms to quantify creative ability in the SZ-spectrum. Examples include investigations looking verbal (Folley and Park, 2005) and visual (Abraham et al., 2005) creativity, but to our knowledge there has been no study of visuospatial creativity, requiring mental imagery abilities, in SZ. As mental imagery is one of the few cognitive functions found to be enhanced in SZ, it may hold the key to uncovering an enhancement in creative functioning in patients with SZ and not simply schizotypes or relatives.

The goal of the current investigation was to help reconcile the deficits and enhancements that have been previously reported in the cognitive profile of SZ patients. We sought to accomplish this broad goal by specifically examining visuospatial mental imagery manipulation ability and its relationship with SWM in SZ patients compared with demographically matched HC. Given that HC show strong positive correlation between mental imagery and SWM, we wanted to further investigate the dissociation between these two cognitive processes in SZ. Finding evidence for this hypothesized MI/SWM dissociation in the current SZ sample would further implicate frontoparietal connectivity abnormalities as a neural marker that may be important for both SZ and creative cognition.

## Methods

### Participants

Eighteen medicated outpatients with schizophrenia (SZ) were recruited from a private psychiatric facility in Nashville, TN. Diagnoses were confirmed using the structured clinical interview for DSM-IV (SCID-IV; First et al., 2002a,b). Eighteen HC participants, with no history of DSM-IV Axis I disorder as confirmed by the SCID-IV in themselves or their families, were recruited from the same city. The two groups were matched for age, sex, handedness (Oldfield, 1971), and premorbid IQ. Since the two groups were not matched for years of education (see Table 1), we used education as a covariate in all between-group analyses. Exclusion criteria for both groups were substance use within the past 6 months, neurological disorders, or history of head injury. Written informed consent was obtained from all participants after they were given a complete description of the study. Participants were paid for their time.

**Table 1:** **Demographic and clinical characteristics of the participants**.

**Demographic variable**	**Schizophrenia patients**	**Healthy controls**	**SZ patients vs. Controls**	
			**Test statistic**	***p*-value**
Age	*M* = 40.1 *SD* = 9.4	*M* = 41.3 *SD* = 8.0	*t*_(34)_ = 0.42	0.6761
Sex	5 females, 13 males	8 females, 10 males	χ^2^_(1)_ = 1.08	0.2979
Handedness	15 right, 3 non-right	17 right, 1 non-right	χ^2^_(1)_ = 1.172	0.2791
Edinburgh laterality	*M* = 56 *SD* = 52.5	*M* = 78.8 *SD* = 46.8	*t*_(34)_ = 1.4	0.1841
Premorbid IQ (NART)	*M* = 103.3 *SD* = 11.77	*M* = 103.27 *SD* = 6.75	*t*_(34)_ = 0.20	0.8416
Years of education	*M* = 13.3 *SD* = 2.2	*M* = 15.1 *SD* = 1.9	*t*_(34)_ = 2.50	0.0174^*^
SPQ (total)	*M* = 39 *SD* = 19	*M* = 12 *SD* = 9	*t*_(28)_ = −5.25	<0.0001^*^
PDI (total)	*M* = 115 *SD* = 73	*M* = 38 *SD* = 27	*t*_(34)_ = −4.15	0.0002^*^
PDI (distress)	*M* = 33 *SD* = 23	*M* = 7 *SD* = 4	*t*_(34)_ = −4.49	<0.0001^*^
PDI (preoccupation)	*M* = 33 *SD* = 22	*M* = 11 *SD* = 8	*t*_(34)_ = −3.87	0.0005^*^
PDI (conviction)	*M* = 39 *SD* = 23	*M* = 16 *SD* = 11	*t*_(34)_ = −3.81	0.0006^*^
PDI (paranoia)	*M* = 52 *SD* = 39	*M* = 17 *SD* = 17	*t*_(34)_ = −3.52	0.0012^*^
PDI (thought disturbance)	*M* = 8.4 *SD* = 11	*M* = 0.3 *SD* = 1.2	*t*_(34)_ = −3.21	0.0029^*^
PDI (catastrophic thought broadcast)	*M* = 10.1 *SD* = 9.5	*M* = 1.5 *SD* = 3.5	*t*_(34)_ = −3.59	0.0010^*^
PDI (ideation of reference)	*M* = 10.0 *SD* = 10.7	*M* = 2.3 *SD* = 4.2	*t*_(34)_ = −2.82	0.0079^*^
BPRS (total)	*M* = 16.5 *SD* = 9.6			
SAPS (total)	*M* = 16.7 *SD* = 10.3			
SANS (total)	*M* = 27.5 *SD* = 15.8			
CPZ-EQ (mg/kg/day)	*M* = 436.9 *SD* = 387.4			
Duration of illness (years)	*M* = 20 *SD* = 9			
Number of hospitalizations	*M* = 12 *SD* = 24			

* *p < 0.05*.

### Design and procedure

#### Clinical measures

Current symptom ratings for SZ patients were assessed on the day of testing using the Brief Psychiatric Rating Scale (BPRS; Overall and Gorham, 1962), the Scale for the Assessment of Positive Symptoms (SAPS; Andreasen, 1984), and the Scale for the Assessment of Negative Symptoms (SANS; Andreasen, 1983). Symptom severity statistics and chlorpromazine equivalent dose (CPZ-EQ; Andreasen et al., 2010) are reported in Table 1. All participants completed the Peters et al. Delusions Inventory (PDI; Peters et al., 2004), a 21-item self-report questionnaire assessing distress, preoccupation, and conviction associated with delusional ideation across nine subscales, including paranoia, thought disturbance, catastrophic thought broadcast, and ideation of reference. All HC and a subset of SZ patients (*n* = 12) completed the Schizotypal Personality Questionnaire (SPQ; Raine, 1991), a 74-item self-report questionnaire assessing schizotypal personality. The SPQ scores reported by healthy participants are typical of the general population (see Raine, 1991).

#### Spatial working memory (SWM) task

SWM ability was assessed with a spatial delayed response task (DRT) (see Park et al., 1999) in all participants with the exception of one SZ. The spatial DRT used in the current study is a very simple, straightforward measure of SWM modeled after single-cell recording studies of non-human primates (Funahashi et al., 1991). During this task, participants were presented with a small target circle for 400 ms, and after a delay of 10 s, were asked to select the remembered location of the target. The task consisted of 48 trials, and the entire task lasted an average of 30 min for each participant. Total proportion of correct trials was quantified as SWM accuracy. Based on previous literature showing that SZ show impaired SWM ability (e.g., Park and Holzman, 1992), we predicted that SZ would exhibit decreased accuracy compared to HC, which would be indicative of impaired maintenance processes in SWM ability.

#### Raven's standard progressive matrices (RPM)

Visuospatial intelligence was assessed with Raven's Standard Progressive Matrices (RPM) (Raven et al., 2003). Importantly, the stimuli are present throughout the task, thus reducing the demand on working memory resources for successful completion of each matrix and instead requiring the manipulation of mental imagery without a working memory maintenance component. Each participant was given unlimited time to complete the 60 matrices and the experimenter noted how long each participant took to complete the task (in minutes), in addition to the standard accuracy scoring guidelines (i.e., raw proportion of accurate responses converted to normed percentile ranks). We hypothesized that patients with schizophrenia would show lower levels of visuospatial intelligence than HC. Thus, we predicted that patients with schizophrenia would be significantly less accurate than HC on RPM and would take significantly longer time to solve the matrices.

#### Paper folding test (PFT)

Visuospatial transformation ability was assessed with the Paper Folding Test (PFT) (ETS, 1962; see also Ekstrom et al., 1976), which requires transformation of mental imagery for successful completion rather than actual paper folding ability (e.g., such as origami). The 20 multiple-choice trials were divided into two test parts, which were each constrained to 3 min for participants to reach as many solutions as possible (out of the maximum 10 per test section). Each correct answer received 1 point, while incorrect answers penalized the participants' scores by subtracting 0.2 points from their total. Skipped items yielded a net score of zero. Given the working memory demands and time constraints inherent in the administration of the PFT, we predicted that SZ patients would perform worse than HC.

#### Jigsaw puzzle task (JPT)

Mental imagery manipulation was assessed with a jigsaw puzzle task (JPT) similar to Richardson and Vecchi's (2002) paradigm. The task began with an instructional demonstration and three practice trials. The experimental trials consisted of 15 different jigsaw puzzles: five 4-fragment puzzles (chair, watering can, iron, baby carriage, telephone), followed by five 6-fragment puzzles (lamp, dresser, shoe, watch, bicycle), followed by five 9-fragment puzzles (teapot, toaster, TV, briefcase, motorcycle). The puzzle images were selected to represent each possible combination of complexity level [based on Snodgrass and Vanderwart (1980) norms, as reported in Richardson and Vecchi (2002)] by fragmentation level. Each participant received the exact same order of JPT stimuli and completed two questionnaires in order to assess comprehension of instruction, enjoyment of task, previous puzzle experience, and self-reported strategies. Finally, participants flipped through a binder of enlarged pictures of the completed jigsaw puzzle images used in the JPT to rate level of complexity, familiarity, and image agreement.

**Figure 1:**
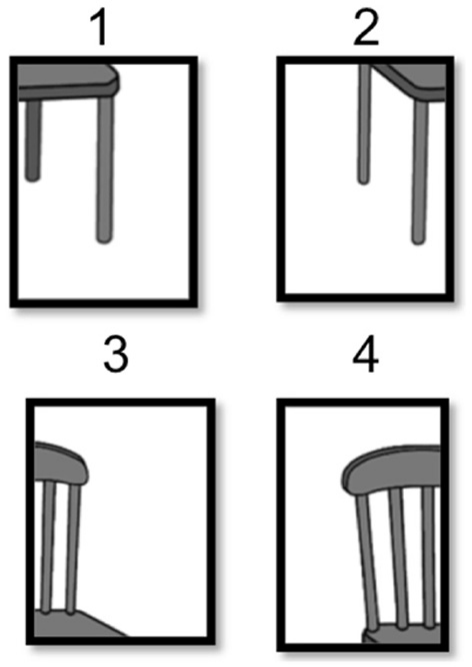
**An example of stimuli in the jigsaw puzzle task**.

Prior to seeing the puzzle pieces, each participant was asked to visualize a certain object before they were presented with a scrambled, fragmented puzzle of the object they were previously instructed to visualize. The participants were given an answer sheet with the exact same grid as the puzzle and were instructed to fill in the numbers of the corresponding puzzle pieces to determine the correct orientation of the puzzle (see Figure 1). Each participant was instructed that they had 3 min to complete each puzzle, but to focus more on accuracy than timing. If a participant went over the 3-min maximum on any particular puzzle, the trial was marked as incorrect. Accuracy (i.e., proportion of correctly placed puzzle pieces) and completion time were recorded for each of the 15 jigsaw puzzles and collapsed across fragmentation. We predicted that SZ would show impaired performance relative to HC given their established impairments in SWM abilities (i.e., slower and less accurate).

#### Line bisection (LB) test

Spatial neglect was assessed with a standard paper-and-pencil version of the line bisection (LB) task (Schenkenberg et al., 1980). Participant's LB's were scored by measuring how many millimeters each drawn bisection was away from the actual center of the of the line. Experimenters noted the magnitude of the deviation from each of the nine trials, in addition to whether or not each deviation was on the left or right side of the actual center of the line. Bias scores were calculated by counting the number of left deviations and number of right deviations to calculate the index scores (i.e., number of right deviations minus the number of left deviations). Then we computed the sum of the left deviations and the sum of the right deviations and used the following formula to calculate bias: [(sum of right deviations/number of right deviations)-(sum of left deviations/number of left deviations)].

Previous research utilizing the LB task has found that spatial neglect is associated with lesions in the right parietal lobule (e.g., Mort et al., 2003; Vandenberghe et al., 2005), right superior temporal gyrus (Karnath et al., 2001), while others have found that neglect results from disconnections in the frontoparietal pathways (He et al., 2007). Based on previous literature implicating parietal abnormalities in SZ in general (Torrey, 2007 for a review) and spatial neglect in particular (Cavezian et al., 2006), we predicted that SZ would demonstrate greater magnitude of LB biases compared with HC (i.e., more right biases). Furthermore, since previous neuroimaging research has demonstrated a relationship between passivity delusions and right parietal abnormalities (Maruff et al., 2005), we predicted that right LB deviations would be related to delusional ideation as quantified by the Peters' et al. Delusions Inventory (PDI).

## Results

A MANCOVA was used to compare group mean performances on the battery of visuospatial tasks, using education as a covariate. The multivariate result was significant for group, Pillai's Trace = 0.560, *F* = 4.725, *df* = (7, 26), *p* = 0.002. The univariate *F*-tests are summarized in Table 2. Correlations among measures are outlined in Table 3.

**Table 2:** **Summary of between-group analyses**.

	**SZ (*n* = 18)**	**HC (*n* = 18)**	**SZ vs. HC**				
			***F***	***p***	**η^2^**	**Power**	**Result**
Spatial DRT accuracy (% correct)	*M* = 85.75 *SD* = 11.1	*M* = 93.92 *SD* = 8.00	4.381	0.044	0.120	0.528	SZ < HC
Raven's matrices accuracy (% rank)	*M* = 29.1 *SD* = 19.7	*M* = 38.83 *SD* = 27.3	0.311	0.581	0.009	0.084	SZ = HC
Raven's matrices time (minutes)	*M* = 37.55 *SD* = 16.5	*M* = 39.88 *SD* = 13.2	0.129	0.721	0.004	0.064	SZ = HC
Paper folding test accuracy (% correct)	*M* = 29.6 *SD* = 1.9	*M* = 36.7 *SD* = 3.1	0.015	0.903	<0.001	0.052	SZ = HC
Jigsaw puzzle task accuracy (% correct)	*M* = 89.53 *SD* = 10.2	*M* = 79.18 *SD* = 16.1	9.307	0.004	0.220	0.842	SZ > HC
Jigsaw puzzle task time (seconds)	*M* = 45.84 *SD* = 16.8	*M* = 62.09 *SD* = 27.4	10.27	0.003	0.237	0.875	SZ < HC
Jigsaw puzzle task imagery agreement	*M* = 3.95 *SD* = 0.78	*M* = 4.20 *SD* = 0.59	2.371	0.133	0.067	0.321	SZ = HC
Jigsaw puzzle task familiarity ratings	*M* = 4.45 *SD* = 0.607	*M* = 4.65 *SD* = 0.45	1.736	0.197	0.050	0.249	SZ = HC
Jigsaw puzzle task complexity ratings	*M* = 2.85 *SD* = 0.92	*M* = 3.24 *SD* = 0.88	0.837	0.367	0.025	0.144	SZ = HC
Line bisection task magnitude of bias	*M* = 0.779 *SD* = 2.3	*M* = −2.01 *SD* = 2.9	9.678	0.004	0.227	0.855	SZ > HC
Line bisection task # right deviations	*M* = 4.44 *SD* = 1.85	*M* = 2.27 *SD* = 2.02	6.493	0.016	0.164	0.696	SZ > HC
Line bisection task # left deviations	*M* = 3.72 *SD* = 1.87	*M* = 5.72 *SD* = 2.21	6.927	0.013	0.173	0.724	SZ < HC

^*^Only 17 SZ patients participated in the spatial delayed response task (DRT).

**Table 3:** **Spearman's correlation matrix**.

	**SWM accuracy**	**Raven's % rank**	**Raven's time**	**PFT accuracy**	**JPT accuracy**	**JPT time**	**LB bias**	**PDI total**	**BPRS total**	**SAPS total**	**SANS total**
SWM accuracy											
Raven's % rank	SZ: 0.57^*^ HC: 0.61^*^										
Raven's time	SZ: 0.18 HC: −0.42	SZ: 0.01 HC: −0.10									
PFT accuracy	SZ: 0.08 HC: 0.59^*^	SZ: 0.64^*^ HC: 0.70^*^	SZ: −0.29 HC:−0.29								
JPT accuracy	SZ: 0.16 HC: 0.64^*^	SZ: 0.167 HC: 0.77^*^	SZ: −0.23 HC: −0.32	SZ: 0.01 HC: 0.74^*^							
JPT time	SZ: 0.03 HC: −0.74^*^	SZ: −0.16 HC: −0.72^*^	SZ: 0.49^*^ HC: 0.27	SZ: −0.48^*^ HC: −0.72^*^	SZ: −0.46 HC: −0.9^*^						
LB bias	SZ: 0.03 HC: −0.05	SZ: −0.13 HC: 0.15	SZ: −0.13 HC: 0.35	SZ: 0.09 HC: −0.12	SZ: −0.09 HC: −0.06	SZ: −0.167 HC: 0.08					
PDI total	SZ: 0.04 HC: −0.65^*^	SZ: −0.36 HC: −0.23	SZ: 0.008 HC: 0.43	SZ: −0.06 HC: −0.49^*^	SZ: −0.29 HC: −0.37	SZ: 0.14 HC: 0.46	SZ: 0.47^*^ HC: 0.18				
BPRS total	SZ: 0.11 HC: n/a	SZ: 0.04 HC: n/a	SZ: 0.13 HC: n/a	SZ: 0.11 HC: n/a	SZ: −0.15 HC: n/a	SZ: 0.11 HC: n/a	SZ: 0.11 HC: n/a	SZ: 0.48^*^ HC: n/a			
SAPS total	SZ: 0.15 HC: n/a	SZ: 0.12 HC: n/a	SZ: 0.07 HC: n/a	SZ: 0.23 HC: n/a	SZ: −0.18 HC: n/a	SZ: 0.16 HC: n/a	SZ: 0.03 HC: n/a	SZ: 0.61^*^ HC: n/a	SZ:0.76^*^ HC: n/a		
SANS total	SZ: −0.18 HC: n/a	SZ: 0.12 HC: n/a	SZ: −0.01 HC: n/a	SZ: 0.28 HC: n/a	SZ: 0.11 HC: n/a	SZ: −0.19 HC: n/a	SZ: −0.09 HC: n/a	SZ: −0.05 HC: n/a	SZ: 0.52^*^ HC: n/a	SZ: 0.05 HC: n/a	

* *p < 0.05*.

Since both groups performed poorly on the PFT (see Table 2), the lack of group difference may be due to a floor effect, masking the variance necessary to detect significant differences between groups. Thus, we conducted further ANCOVA analyses to compare performance between the two groups in terms of total number of errors, *F*_(1,33)_ = 1.268, *p* = 0.268, and total number of skipped items, *F*_(1,33)_ = 1.493, *p* = 0.230, to ensure that this null finding was not the result of different strategies between the two groups (i.e., choosing to skip certain questions). However, the results from these additional tests indicate that there were no group differences on the PFT, providing evidence for spared, or intact ability for patients with schizophrenia to transform visuospatial imagery.

A chi-square contingency table was created and a subsequent Fisher's Exact Test was conducted to compare previous puzzle experience between SZ and HC. There was a trend-level difference [χ^2^_(1)_ = 3.7, *p* = 0.06] in levels of self-reported experience with puzzles between SZ and HC. JPT accuracy was found to be unrelated to symptoms ratings (see Table 3 below), duration of illness (*r*_*s*_ = −0.1533, *p* = 0.5436), or CPZ equivalent dosage (*r*_*s*_ = 0.1597, *p* = 0.5404) in SZ. For HC, spatial DRT accuracy and JPT accuracy were positively correlated (*r*_*s*_ = 0.64, *p* = 0.0043), but were essentially unrelated in SZ (*r*_*s*_ = 0.16, *p* = 0.5231).

Among SZ, LB bias was positively correlated with total scores of the PDI (*r*_*s*_ = 0.47, *p* = 0.04), thought disturbance subscale (*r*_*s*_ = 0.51, *p* = 0.03), catastrophic thought broadcast subscale (*r*_*s*_ = 0.49, *p* = 0.04), and ideation of reference subscale (*r*_*s*_ = 0.53, *p* = 0.02). LB bias was positively correlated with distress associated with delusional ideation (*r*_*s*_ = 0.50, *p* = 0.03) and preoccupation with delusions (*r*_*s*_ = 0.48, *p* = 0.04) in SZ patients. In HC, PDI distress was positively correlated with LB bias (*r*_*s*_ = 0.50, *p* = 0.03) and number of right deviations (*r*_*s*_ = 0.48, *p* = 0.03). Further, the number of right deviations was positively related to the paranoia subscale of the PDI (*r*_*s*_ = 0.53, *p* = 0.02).

## Discussion

Mental imagery is intimately linked to memory (Baddeley and Andrade, 2000), as depicted by the visuospatial sketchpad component of the dominant theory of working memory (Baddeley, 1992; Logie, 1995). Both working memory and imagery rely on the maintenance and manipulation of internal representations (Kosslyn, 1980; Slotnick et al., 2012). Indeed, generating and inspecting internal representations of stimuli are known to aid memory performance (Gur and Hilgard, 1975; Berger and Gaunitz, 1979), and studies examining the role of visuospatial imagery in memory have mostly reported a strong association (Marks, 1973; Gur and Hilgard, 1975; Baddeley and Andrade, 2000). This is not surprising given the overlap between mental representation imagery and working memory processes and the neural circuits mediating them (Kosslyn, 1980; Baddeley and Andrade, 2000; Harrison and Tong, 2009; Kalkstein et al., 2011; Slotnick et al., 2012; Albers et al., 2013).

Previous research suggests that SZ patients consistently demonstrate an impaired ability to maintain mental representations, despite a superior ability to generate, inspect, and manipulate mental imagery (Thakkar and Park, 2012; Matthews et al., 2013). Our hypotheses were confirmed with the finding that SZ were significantly less accurate than HC on the spatial DRT. Thus, results from the current study demonstrate that the current SZ sample was significantly impaired on SWM maintenance compared to demographically matched HC. This result indicates that our sample of SZ is comparable to previous research investigating SWM in SZ, allowing for ease of generalization of the current results. Further, these results indicate this deficit may be due to impaired maintenance rather than manipulation since SZ showed intact or enhanced performance on SWM tasks that did not require maintenance, which is consistent with previous research (e.g., Thakkar and Park, 2012; Matthews et al., 2013).

SZ and HC showed equal performance on the PFT, indicating spared visuospatial transformation ability in SZ. Furthermore, SZ and HC showed equal accuracy and completion time for RPM. The fact that we found no significant group differences between SZ and HC on RPM indicates that the two groups were matched for visuospatial intelligence. This is an important point since the two groups were recruited with the intent to be matched for premorbid intelligence without accounting for current intelligence in our SZ patient sample. Thus, equal performances on Raven's indicates that these two groups were matched on relevant levels of intelligence, suggesting that any group differences that we found on the other three administered tasks were due to variability inherent between the two groups not confounded by differences in IQ.

Consistent with previous literature, we found increased LB bias magnitude (in general) and greater sum of right deviations in particular, in patients with SZ compared to HC. This finding provides further evidence implicating the right parietal cortex as an important site for neural anomalies in the schizophrenia-spectrum. Consistent with recent reports implicating right LB bias as a potential marker for an endophenotype candidate for the schizophrenia-spectrum, we also found behavioral evidence for possible right parietal abnormalities in patients with SZ. However, LB bias in both SZ and HC, particularly magnitude of right deviations, was strongly related to delusional ideation in the current sample. Indeed, previous neuroimaging research has demonstrated a relationship between passivity delusions and right parietal abnormalities (Maruff et al., 2005). Therefore, it is unclear whether or not LB bias and the underlying right parietal abnormalities should be treated as an endophenotype latent for SZ liability, or if it is more directly related to the development of SZ symptomatology (i.e., delusions).

SZ performed significantly better than HC on the JPT. Specifically, SZ successfully completed the jigsaw puzzles in less time, and with fewer errors, than demographically matched HC. Further, SZ's JPT performance was unrelated to symptom severity, medication dosage, or illness duration. Consistent with Sack et al. (2005), the current results indicate that enhanced mental imagery was unrelated to current psychopathology, providing support for the hypothesis that enhanced mental imagery could be a trait marker for the schizophrenia-spectrum.

SZ did not differ from HC in their post-task puzzle ratings. The fact that we found no significant group differences between SZ and HC on the complexity and familiarity ratings indicates that there were no prior biases that may have placed SZ at a greater advantage for solving the puzzles faster and more accurately than controls (other than superior imagery manipulation ability). The fact that there was no group difference on the image agreement ratings could mean one of two things: either the patients were able to use a strategy other than superior imagery generation to outperform HC on these tasks, or that the JPT used in the current study is a more sensitive measure of mental imagery than self-report methods. It is unclear from the results of this study which explanation is more plausible. However, at least one group of studies also found similar results suggesting superior mental imagery abilities via behavioral methodology, but not through self-reported methods (e.g., Matthews et al., 2013). Thus, behavioral methods might be more sensitive or self-reported measures of mental imagery might be tapping into a different process than behavioral methods.

Mental imagery and SWM abilities were strongly correlated in HC. In contrast, these two functions are dissociated in SZ, which may indicate abnormal connectivity of the frontoparietal network. Previous research has demonstrated that the parietal and frontoparietal networks may be important for creative cognition (e.g., Gaser and Schlaug, 2003), and visuospatial creativity in particular (e.g., Seeley et al., 2008; Kowatari et al., 2009). Creative professions are more common in the relatives of patients with schizophrenia, bipolar disorder, anorexia nervosa, and, to some extent, autism (Kyaga et al., 2012). Furthermore, creativity has been linked to schizophrenia empirically (e.g., Keefe and Magaro, 1980; Folley, 2006) and in the context of modernism (Sass, 2001). The current results suggest that patients with SZ are unable to recruit their superior mental imagery ability during SWM tasks (i.e., they are not utilizing their visuospatial sketchpad, despite its superiority compared to HC). Understanding the connections between these dissociated cognitive mechanisms might be the key to understanding how abnormal cognitive processes contribute to the development of positive symptoms that are characteristic of patients with SZ. Similarly other cognitive processes such as visuospatial creativity might also be dissociable from mental imagery or working memory depending on the nature of the task used, leading to an incomplete understanding of the phenomenon.

The current results also suggest a potential cognitive remediation strategy for SZ. In other words, superior mental imagery manipulation ability in SZ could be leveraged to support working memory during cognitive training. This hypothesis is supported by preliminary evidence in the current study when one considers the discrepancy between presence vs. absence of group differences between the PFT and the JPT. Specifically, participants were repeatedly instructed to utilize mental imagery when solving the jigsaw puzzles, which is contrasted with the PFT in which participants were only instructed to use imagery during the initial instructions. Future research should explicitly test the hypothesis that a failure of connection between mental imagery and working memory regions could underlie some of the cognitive deficits and psychotic symptoms observed in SZ patients.

One potential limitation of the current study was that the two groups were not matched for years of education or previous puzzle experience. In detail, HC had significantly more years of education than SZ, in addition to higher levels of previous experience with jigsaw puzzles, particularly in the most recent 5 years prior to testing. Specifically, HC reported slightly more recent experience with jigsaw puzzles as a result of playing similar games with their children. However, despite greater experience with jigsaw puzzles and higher education level, the HC group did not perform better than SZ on the mental imagery tasks. Future research should attempt to match these demographic characteristics to help ensure that any group differences are not likely due to extraneous confounds. Still, as these discrepancies favor HC over SZ in the present study (i.e., HC had more education), the superior performance of SZ cannot be explained by these variables and could even be interpreted as a potential strengthening factor in light of the current results. In other words, even with less puzzle experience and a significant deficit in educational training, SZ still managed to outperform HC on the JPT, presumably by utilizing their superior visuospatial imagery manipulation abilities.

In sum, the results from the current investigation utilizing behavioral spatial cognition tasks suggest that in addition to showing evidence of parietal abnormalities, patients with SZ demonstrate impaired maintenance component of SWM, intact visuospatial intelligence and transformation, and enhanced visuospatial manipulation. SWM and mental imagery were positively correlated in HC but were essentially unrelated in SZ. This pattern of results is consistent with previous literature (Matthews et al., 2013) and provides further evidence that SWM and imagery manipulation ability are dissociated in SZ. Experimental tasks must be developed to assess visuospatial creativity more accurately, without the confounds of working memory deficits in patients with schizophrenia. Most important, however, is the need for neuroscience methodology to be utilized to understand the importance of mental imagery in creative cognition in both healthy and clinical populations.

### Conflict of interest statement

The authors declare that the research was conducted in the absence of any commercial or financial relationships that could be construed as a potential conflict of interest.
